# Plasmon-Enhanced Visible and Near-Infrared Photodetection with Gold Nanorods UCNPs/MoS_2_ Hybrid Device

**DOI:** 10.3390/ijms26083480

**Published:** 2025-04-08

**Authors:** Haitao Wei, Bowen Lv, Mengya Zhang, Xiangzhe Zhang, Xingheng Yan, Junhao Cai, Yaping Yang, Tongcheng Yu

**Affiliations:** 1 College of Advanced Interdisciplinary Study, National University of Defense Technology, Changsha 410073, China; weihaitao0903@nudt.edu.cn (H.W.); lvbowen22@nudt.edu.cn (B.L.); zhangmengya23@nudt.edu.cn (M.Z.); xiangzhe.cheung@gmail.com (X.Z.); jh.cai@nudt.edu.cn (J.C.); 2 College of Aerospace Science and Engineering, National University of Defense Technology, Changsha 410073, China; 17801016168@163.com (X.Y.); ypyang0916@outlook.com (Y.Y.)

**Keywords:** plasmon, Au NRs, UCNPs, MoS_2_, photodetector

## Abstract

The near-infrared photodetection of monolayer MoS_2_ can be achieved using upconverted nanoparticles (UCNPs). Herein, we demonstrated that gold nanorods (Au NRs) further enhanced the near-infrared photoresponsivity of a hybrid device via the surface plasmon enhancement of the localized field. We synthesized a three-layer device comprising Au NRs, UCNPs (NaYF_4_:Yb^3+^, Er^3+^), and monolayer MoS_2_, and examined its photoelectric characteristics using laser irradiation with varying power densities at 980 nm, the excitation wavelength of UCNPs. Compared with a device without Au NRs, the photoelectric response of the three-layer device was greatly improved at 5 V bias, and photoresponsivity was increased at visible wavelengths (450, 532, and 635 nm). This study contributes to the knowledge of two-dimensional materials for the development of hybrid photoelectronic devices.

## 1. Introduction

Photodetectors, as crucial components in the information perception field, transform optical signals into electrical impulses. Infrared (IR) photoelectric detectors, which visualize IR signals carrying the radiation characteristics of objects via photoelectric conversion and electrical signal processing, are extensively adopted in a variety of applications, including laser radar, night vision imaging, optical fiber communication, and laser distance measurement [1,2,3]. The traditional semiconductor material, silicon (Si), has drawbacks such as low light absorption within the IR band and excessive dark current owing to its indirect bandgap characteristics. However, photodetectors based on InGaAs and HgCdTe crystals are sophisticated, costly, and usually require refrigeration, which cannot meet the needs of miniaturization [4,5].

Compared with traditional commercial materials, two-dimensional (2D) materials have unique advantages in terms of size and photoelectric response range, providing new possibilities for the development of highly integrated and wide-spectrum photodetectors [4]. In recent years, a novel manufacturing strategy for near-infrared (NIR) photodetectors based on 2D materials was proposed using graphene and black phosphorus, broad-spectrum 2D materials that are sensitive to NIR radiation [6,7,8,9]. Graphene is a very thin, flexible, and durable material that can be fabricated on a large scale, with an ultrahigh carrier mobility of approximately 100,000 cm^2^·V^−1^·s^−1^ [10,11,12]. However, the use of graphene in transistors, which require a clear ON/OFF state, is challenging owing to its zero-bandgap properties [13]. Black phosphorus also has very high carrier mobility, but rapid oxidation in air greatly limits its use [6,14,15]. WSe_2_ and MoS_2_, members of the layered transition metal dichalcogenide (TMD) family (structure MX_2_, where M = Mo, W, Nb, Ta, Ti, and Re; X = S, Se, and Te), which has a hexagonal single-layer structure, shows many special electrical and optical properties, and has become a hotspot in the field of optoelectronic devices. However, monolayer WSe_2_ has a higher bandgap and lower carrier mobility than MoS_2_ [16]. MoS_2_ has garnered considerable interest among researchers as a material for photodetectors owing to its high electron mobility and outstanding ON/OFF current ratio [17,18]. The direct bandgap of single or few layers of MoS_2_ makes it a promising material for photoelectric applications. However, MoS_2_ only exhibits substantial photoresponsivity within a limited spectral band [19] in the visible (VIS) range, limiting its use in multiband detection applications such as imaging, remote sensing, and spectral analysis.

Upconversion nanoparticles (UCNPs) are a special type of rare earth element nanoparticles. Owing to their unique two-photon and multiphoton processes, they can absorb low-energy light to produce high-energy light, achieving the “upconversion” of light [20,21]. Typical UCNPs based on NaYF_4_ can absorb NIR light (excitation wavelength range of 808–1550 nm) to produce VIS light [22]. Recent studies have combined UCNPs with TMDs, achieving simultaneous photoelectric response within the response range of TMDs (VIS light) and the excitation range of UCNPs (NIR) [23,24,25,26]. This method is simple and suitable for industrial needs. However, the photoelectric response of UCNPs/TMDs within the NIR band requires improvement owing to the low upconversion efficiency of UCNPs. The plasmon resonance caused by VIS or NIR radiation on the surface of metal nanoparticles generates a local electromagnetic field that is several orders of magnitude stronger than the incident light field. Notably, gold nanorods (Au NRs) have recently emerged as a promising material for photoelectric applications owing to their anisotropic form and tunable plasmonic characteristics [27,28]. The surface plasmon resonance wavelengths of Au NRs can be adjusted from VIS (550 nm) to NIR (1550 nm) depending on the particle diameter ratio [29]. Photofield-effect transistors made of semiconductor single-wall carbon nanotubes (SWCNTs) and non-covalently connected gold nanoparticles [30,31], metal nanoparticles or plasma nanocavities and WSe_2_ [32] showed good response performance.

Here, we describe a novel strategy for NIR detection by simply combining Au NRs, UCNPs, and MoS_2_. As absorbers of NIR light, UCNPs can efficiently convert IR light into VIS light. NaYF_4_:Yb^3+^/Er^3+^ was selected as the NIR absorber, which exhibits a substantial absorption peak at approximately 980 nm. The plasmonic enhancement of noble metal nanomaterials is an effective method to improve the luminescence intensity of UCNPs [27,33,34,35]. Thus, we chose Au NRs as NIR radiation intensifiers to enhance the absorbed radiation energy of UCNPs via a plasmon-enhancing effect, thereby boosting the upconversion of UCNPs. MoS_2_ acts as a receiver of the upconverted radiation energy. The fabricated hybrid device (Au NRs/UCNPs/MoS_2_) exhibited distinct enhancements compared to a device without Au NRs (UCNPs/MoS_2_) within the VIS and NIR (980 nm) spectrum.

## 2. Results and Discussion

**Figure 1:**
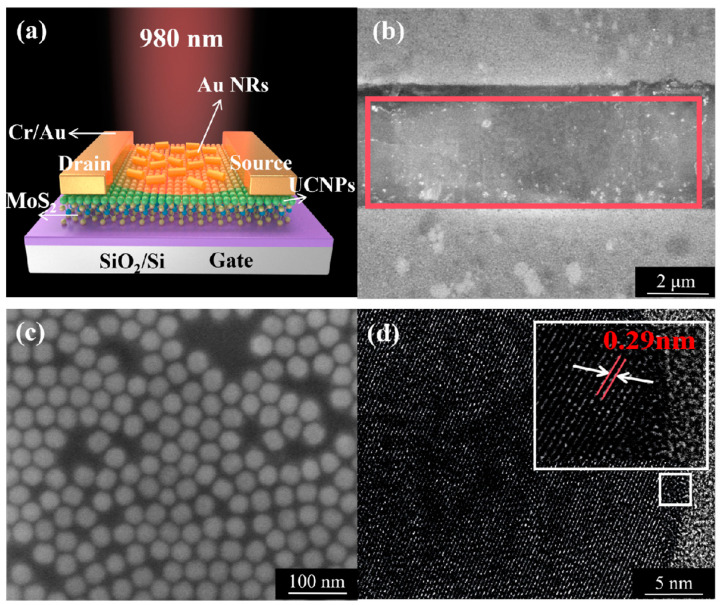
(**a**) Cross-sectional image of the Au NRs/UCNPs/MoS_2_ hybrid device on a SiO_2_/Si substrate with Cr/Au contacts. (**b**) SEM image of the Au NRs/UCNPs/MoS_2_ hybrid device on a SiO_2_/Si, the dashed line depicts the monolayer MoS_2_ flake. (**c**) SEM image of UCNPs after spin-coating. (**d**) TEM image of UCNPs with a lattice spacing of 0.29 nm.

Figure 1a depicts a schematic of the Au NRs/UCNPs/MoS_2_ hybrid device. First, a dispersion of UCNPs (NaYF_4_:Yb^3+^/Er^3+^) in cyclohexane was spin-coated onto monolayer MoS_2_ on a Si substrate with a 300 nm SiO_2_ layer. The Au NR solution was then dropped onto the UCNPs/MoS_2_ hybrid device and allowed to dry. A scanning electron microscopy (SEM) image of the Au NRs/UCNPs/MoS_2_ hybrid device is presented in Figure 1b, displaying MoS_2_ in the middle of the electrode (dashed line), a large number of uniformly sized UCNPs scattered on the surface of the MoS_2_ flake underneath, and the dispersed distribution of Au NRs. As shown in Figure S3a, elemental analysis was performed on samples within the red area, and Si elements in the base were removed. Figure S3c presents the elemental proportions in the red area, revealing Mo and S elements belonging to MoS_2_; Na, Y, F, Yb, and Er elements belonging to UCNPs, the O element belonging to the SiO_2_ substrate; and the Au element belonging to Au NRs. Figure 1c illustrates the uniform distribution of UCNPs on the sample after spin-coating. The atomic force microscopy (AFM) data (Figure S5) indicated that the UCNP film comprised single or double layers. Figure 1d displays the atomic lattice fringes of UCNPs captured by transmission electron microscopy (TEM), revealing a lattice spacing of approximately 0.29 nm, which corresponded to the hexagonal crystal phase, as previously reported [36]. According to the statistical results shown in Figure S4, the UCNP diameter was approximately 43 nm. Au NR morphology was characterized by TEM, as shown in Figure S2a,b, which displays the atomic lattice fringes of Au NRs, revealing a lattice spacing of approximately 0.21 nm. According to the statistical results shown in Figure S2c,d, the Au NR length and cross-sectional diameter were approximately 143 and 26 nm, respectively, with a 1:6 particle size ratio.

**Figure 2:**
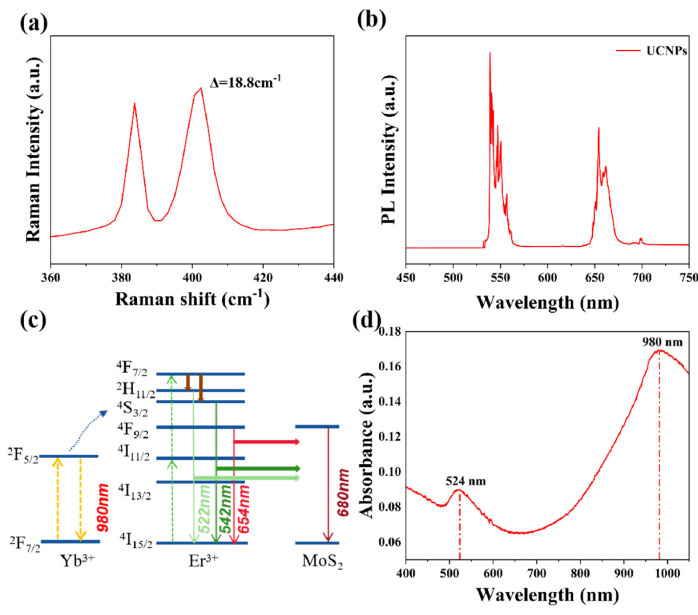
(**a**) Raman spectrum of bare MoS_2_ on SiO_2_/Si, demonstrating the E_2g_ and A_1g_ peaks of MoS_2_. (**b**) PL spectrum of UCNPs with 980 nm excitation (P = 475 W/cm^2^). (**c**) Schematic of the excitation of electrons and energy transfer from UCNPs to MoS_2_ in the hybrid device. (**d**) Absorption spectrum of Au NRs in an aqueous solution.

Raman peaks at 383.7 and 402.5 cm^−1^, shown in Figure 2a, were identified as the E_2g_ and A_1g_ peaks of MoS_2_, respectively. The separation between the two peaks, which was less than 20 cm^−1^, helped prove the monolayer property of MoS_2_ [23,37,38]. As can be seen from Figure S1b, the photoluminescence (PL) peak of MoS_2_ was determined at 680 nm (1.82 eV), which was consistent with the PL peak of monolayer MoS_2_ reported in the literature [17,39]. Figure 2b presents the PL spectrum of UCNPs excited by 980 nm light, with the fluorescence emitted by UCNPs mainly concentrated at 530–560 and 650–670 nm, which could be absorbed by MoS_2_, thus activating the photoresponse of monolayer MoS_2_ within the NIR spectral range. Figure 2c illustrates the mechanism of energy transfer between UCNPs and MoS_2_. NIR radiation at 980 nm can be absorbed by Yb^3+^, causing the transfer of electrons from ^2^F_7/2_ to ^2^F_5/2_. The energy is transmitted to Er^3+^, and electrons are absorbed into the higher energy levels of Er^3+^ via two-photon absorption [7]. Radiation at shorter wavelengths (522, 542, and 654 nm) is released through the transfer processes of ^2^H_11/2_, ^4^S_3/2_, and ^4^F_9/2_→^4^I_15/2_. Thus, the incident radiation at the longer wavelength is upconverted into radiation at shorter wavelengths through UCNPs, and high-energy excitons at shorter wavelengths are absorbed by monolayer MoS_2_, producing the photoresponse. The absorption spectrum of Au NRs is displayed in Figure 2d. Notable absorption peaks were observed at approximately 524 and 980 nm, suggesting the strong surface plasmon-enhanced effect of Au NRs at these wavelengths. When the transverse and longitudinal absorption peaks of gold nanorods align with the emission and absorption wavelengths of upconversion nanoparticles, both the excitation and emission efficiencies of the upconversion nanoparticles can be simultaneously enhanced, thereby intensifying the upconversion luminescence [40]. The UCNPs we used absorbed 980 nm light and emitted fluorescence at 532 nm. Therefore, we chose Au NRs with a 1:6 aspect ratio. The plasmon resonance wavelengths of Au NRs were directly related to the particle aspect ratio, which will be discussed later with COMSOL simulations (version 6.0.318).

**Figure 3:**
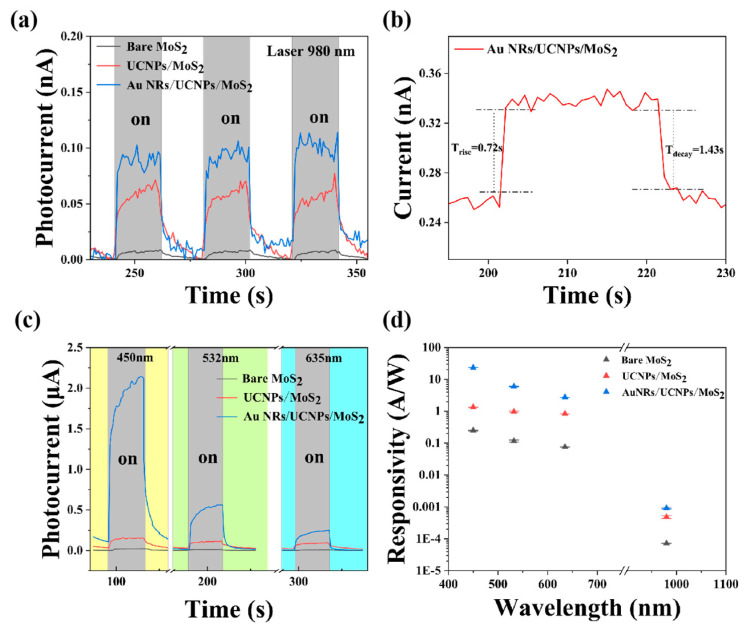
Photoelectronic properties of the Au NRs/UCNPs/MoS_2_ hybrid, UCNPs/MoS_2_ hybrid, and monolayer MoS_2_ photodetectors under 980 nm illumination. (**a**) Switching characteristic curves of the three devices under the same power density of 980 nm laser irradiation (P = 0.13 W/cm^2^). (**b**) Time-resolved photocurrent rise and decay rate of the Au NRs/UCNPs/MoS_2_ hybrid device after 980 nm laser switching on and off under ambient conditions. (**c**) Switching characteristic curves of the three devices under 450, 532, and 635 nm optical excitation with the same power density (P = 0.13 W/cm^2^). (**d**) Photoresponsivity of the three devices under 450, 532, 635, and 980 nm optical excitation with the same power density (P = 0.13 W/cm^2^).

The photoresponses of the Au NRs/UCNPs/MoS_2_ hybrid photodetector, bare MoS_2_, and UCNPs/MoS_2_ hybrid device measured under ambient conditions are presented in Figure 3a. The laser irradiation had a power of 0.13 W/cm^2^ with a spot size of approximately 1.5 mm, and the bias voltage between the source and drain of MoS_2_ was set to 5 V for a relatively large photocurrent. The switching photocurrent (I_ph_) with the light on and off, shown in Figure 3a, is defined as the difference between I_on_ and I_off_. The photocurrent of the bare MoS_2_ under 980 laser irradiation was extremely close to the dark current because the photon energy was less than the bandgap of monolayer MoS_2_, which was insufficient to excite electrons from the valence to conduction bands. This increased dark current may be caused by the photothermal effect. Figure 3a presents the 1.91-times-higher photoresponse of the Au NRs/UCNPs/MoS_2_ device compared to the UCNPs/MoS_2_ hybrid device under 980 nm radiation, which resulted from the plasmon effect of Au NRs on UCNPs. Figure 3b presents the time-resolved photocurrent of the Au NRs/UCNPs/MoS_2_ hybrid device under 980 nm radiation. The rise and decay times were calculated to be 0.72 and 1.43 s, respectively, estimated by the time taken from 10% to 90% of the final values or the inverse. Figure S6 illustrates the dependence of the photocurrent of the Au NRs/UCNPs/MoS_2_ hybrid photodetector on the intensity of the 980 nm laser. The photocurrent increased with increasing intensity of the incident light. This curve was derived from another prepared Au NRs/UCNPs/MoS_2_ hybrid device, where the current and power followed the power formula I_ph_∝P^2^, and the coefficient 2 was associated with complicated processes within the semiconductor, including electron–hole generation, trapping, and recombination [41]. Figure 3c presents the switching characteristics of bare MoS_2_, the UCNPs/MoS_2_ device, and the Au NRs/UCNPs/MoS_2_ device, with the same power density (P = 0.13W/cm^2^) and VIS laser irradiation at 450, 532, and 635 nm. The scale of the photocurrent at these VIS wavelengths was much larger than that under 980 nm radiation, and the enhancement induced by Au NRs was more obvious. The time-resolved photocurrents of the Au NRs/UCNPs/MoS_2_ device under 450, 532, and 635 nm radiation are shown in Figure S5. The rise and decay times of the device at 980 nm, where upconversion occurred, were much shorter than those in the VIS range. This was attributed to the high energy of the VIS light laser, and the continuous photothermal effect led to an increased photocurrent. Under laser irradiation, the photothermal effect will cause the current increase. The general response time of photoconductivity effect is below the order of μs. Therefore, in Figure S5, the slow-growing photocurrent is caused by the photothermal effect of visible light. Since the photothermal effect of visible light is much stronger than that of near-infrared light, it can be seen from the Figure S7 that when there is no laser irradiation, the current level of the device is still higher than the initial current level for a short time, and the photothermal effect will lead to the increase in the dark current of the device. Figure 3d presents the photoresponsivity of the three devices separately exposed to laser irradiation at 450, 532, 635, and 980 nm with the same power density (P = 0.13 W/cm^2^). Photoresponsivity describes the photoelectric conversion capability of the device and can be calculated using the formula R = I_ph_/PS, where P is the incident light power intensity and S is the effective area under illumination. The photoresponsivity of the Au NRs/UCNPs/MoS_2_ device increased by 17.1, 6.2, 3.3, and 1.91 times compared to the UCNPs/MoS_2_ device at 450, 532, 635, and 980 nm, respectively. It can be seen from the first principle that the bandgap of monolayer MoS_2_ is about 1.8 eV (653 nm) [42], so the light energy in the visible band is directly absorbed by MoS_2_, making the device’s performance under visible laser irradiation higher than that of near-infrared light. The results demonstrated that Au NRs greatly enhanced the photoresponse capabilities of MoS_2_ within the VIS spectrum. UCNPs do not absorb VIS light; thus, the increased VIS photoresponse after spin-coating UCNPs might be attributed to repeated reflections of light between UCNPs and MoS_2_, where nanocavities formed [25]. When the device is irradiated by 980 nm laser, the increase in photocurrent is mainly due to photothermal effect and the absorption of visible light by UCNPs upconverted by MoS_2_, and the visible light radiated by UCNPs is enhanced by the plasmonic effect of Au NRs. In addition, Au NRs exhibited a transverse absorption peak at approximately 532 nm, reflecting that the plasmon enhancement effect also occurred under the excitation of VIS light. We compared the performance of our device with other reported photodetectors in Table 1.

**Figure 4:**
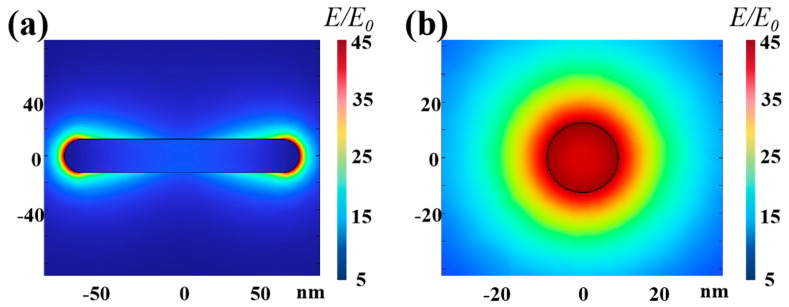
Sectional pattern of light field distribution for Au NRs with a particle length ratio of 6:1 under the wavelengths of 980 nm: (**a**) 980 nm light illumination at the XY-plane; (**b**) 980 nm light illumination at the YZ-plane.

COMSOL simulations were utilized to assess the field intensity distribution of Au NRs when excited by a 980 nm laser. The constructed Au NRs were set with a length and diameter of 150 and 25 nm, respectively. Figure 4a,b presents sectional views of the light field distribution for Au NRs with a 1:6 particle size ratio at the XY- and YZ-planes under 980 nm light illumination. The field strength at both ends of the Au NRs was greatly enhanced, stimulating UCNPs to emit stronger fluorescence within the VIS band.

**Figure 5:**
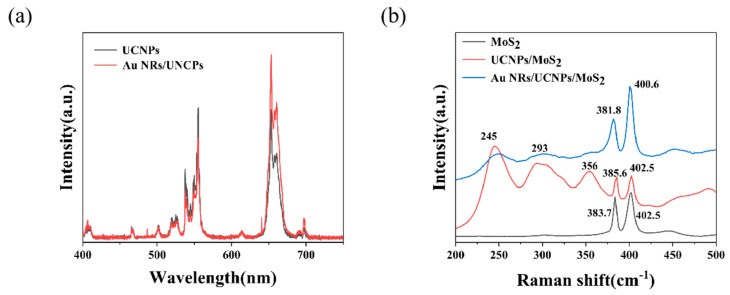
(**a**) PL spectrum of bare UCNPs and Au NRs/UCNPs devices. (**b**) Raman spectrum of the Au NRs/UCNPs/MoS_2_ device, UCNPs/MoS_2_ device, and bare MoS_2_ on SiO_2_/Si, demonstrating the E_2g_ and A_1g_ modes for the three structures.

To further understand the mechanism underlying the enhanced photoresponse of the hybrid device induced by Au NRs, we measured the PL spectrum of bare UCNPs and the Au NRs/UCNPs device when excited by a 980 nm laser (P = 475 W/cm^2^), as shown in Figure 5a. After adding Au NRs, the fluorescence spectrum of UCNPs at approximately 650 nm was substantially enhanced. This was attributed to the surface plasmon enhancement of Au NRs under 980 nm excitation increasing the fluorescence energy emitted by UCNPs. However, the fluorescence peak intensity of UCNPs at approximately 532 nm did not change much or even slightly decreased. As mentioned above, Au NRs also exhibited an absorption peak at 532 nm, and we speculate that the enhanced fluorescence of UCNPs at this wavelength was absorbed by Au NRs. The Raman spectra of bare MoS_2_, the UCNPs/MoS_2_ device, and the Au NRs/UCNPs/MoS_2_ device were further investigated. Figure 5b depicts the characteristic phonon bands of UCNPs at 245, 293, and 353 cm^−1^, which varied depending on the size and structure of the nanoparticle, resembling those observed in previously reported colloidal Yb^3+^ and Er^3+^ codoped NaYF_4_ nanophosphors with a particle size of 47 nm [53,54,55]. The E_2g_ and A_1g_ modes represent the parallel and perpendicular vibrations of MoS_2_, respectively. Interestingly, the E_2g_ and A_1g_ peaks of MoS_2_ were red-shifted after Au NRs were added, revealing n-type doping applied to monolayer MoS_2_. The added Au NRs caused an increase in the electron concentration and strong coupling between phonons and electrons, decreasing the vibration frequency of sulfur atoms, which caused the redshift of the A_1g_ peak, and may indicate the presence of electron tunneling from Au NRs into MoS_2_ with light excitation.

## 3. Methods and Materials

### 3.1. Materials Preparation

The MoS_2_ flake was produced by SixCarbon Technology (Shenzhen, China). The UCNPs (NaYF_4_:Yb^3+^, Er^3+^) were produced by Shaanxi Bangshi Biotechnology Co., Ltd. (Xi’an, China). The Au NRs were produced by Xianfeng Nanomaterial Technology Co., Ltd. (Nanjing, China).

### 3.2. Device Fabrication

Cr/Au was employed for deposition on monolayer MoS_2_ samples, and the channel width between electrodes was 5 μm. The electrodes we used were 50 nm thick of Au covered with a layer of 5 nm Cr. NaYF_4_:Yb^3+^/Er^3+^ was dispersed in cyclohexane, centrifuged at 10,000 r/min for 3 min, and the supernatant was extracted and filtered. The obtained UCNPs solution was treated by ultrasound for 30 min before spin-coating over monolayer MoS_2_ at 4000 r/min for 60 s. Finally, the aqueous Au NR solution was treated by ultrasound for 10 min. Then, the treated aqueous Au NR solution was dropped onto the sample and allowed to air dry.

### 3.3. Characterizations and Measurement

UCNPs, Au NRs, and the samples were examined by TEM (Tecnai G2 F20; FEI Ltd., Hillsboro, OR, USA), SEM (Hitachi, Tokyo, Japan), and AFM (Dimension Icon; Bruker, Karlsruhe, Germany). Raman and PL spectroscopies were performed using a confocal micro-Raman spectrometer system (Renishaw, Wotton-under-Edge, UK) with a 50× objective lens. For photodetection under VIS and NIR lasers, the photocurrent was collected by a semiconductor characterization system (Probe station and 2450 SourceMeter; Keithley Instruments, Solo, OH, USA) at room temperature.

## 4. Conclusions

We demonstrated a novel strategy to enhance the NIR photoelectric response of a UCNPs/MoS_2_ hybrid photodetector using Au NRs with an average diameter and length of 26 and 143 nm, respectively. The photoelectric response of the hybrid device at 980 nm was enhanced by the addition of Au NRs, reaching a maximum photoresponse 1.91 times higher than that of the device without Au NRs (UCNPs/MoS_2_). The enhancement of the device response is mainly due to the photothermal effect, the upconversion of UCNPs, and the plasmonic effect of Au NRs. The change in the PL spectra proved that the upconversion of UCNPs was indeed promoted by Au NRs, resulting in stronger fluorescence of UCNPs, which resulted in enhanced photoresponse. Moreover, the addition of Au NRs enhanced the photoelectric response within the VIS light spectrum, which may be related to the nanocavity effect. In addition, the periodic arrangement of Au NRs and annealed devices is expected to further improve device performance.

## Figures and Tables

**Table 1:** Previous studies of similar PDs included in the study.

Material	R (mA/W)	D* (×10^9^ Jones)	Rise Time (s)	Wavelength (nm)	Ref.
MoS_2_/UCMCs	0.1	0.1	/	980	[43]
Monolayer MoS_2_/UCNPs	10.5	/	7.9	980	[24]
SLG-CNTF device	209	48.7	6.8 × 10^−6^	980	[44]
SWNTs/C_60_ phototransistor	1.94 × 10^4^	1.17	2 × 10^−3^	/	[45]
CNT–ANs (Ag_2_S NPs)	8.3 × 10^3^	17	/	White light	[46]
SnSe_2_/Ag NP: SiO_2_	194.4	/	/	405	[47]
Ag-rGO	1.423 × 10^4^	717	/	682	[48]
Ag NPs/formamidinium-based perovskite	1.03	3.7 × 10^3^	/	/	[49]
Au NPs/ReS_2_	1.3	7.27 × 10^2^	0.2	780	[50]
GaAs NW/AuNPs/	3.047 × 10^3^	/	/	532	[51]
Au NWs/SiNHs	15	/	0.339	650	[52]
Au NRs/UCNPs/MoS_2_	2.3 × 10^4^	/	7.8	450	This work
Au NRs/UCNPs/MoS_2_	0.92	/	0.72	980	This work

## Data Availability

Data is contained within the article and Supplementary Materials.

## Supplementary Materials

The following supporting information can be downloaded at: https://www.mdpi.com/article/10.3390/ijms26083480/s1.
