# Supplementary material for: Plasmon-Enhanced Visible and Near-Infrared Photodetection with Gold Nanorods UCNPs/MoS2 Hybrid Device
Source: Int J Mol Sci. 2025 Apr 8;26(8):3480. doi: 10.3390/ijms26083480 (PMC12026974; doi:10.3390/ijms26083480)
Supplement: Supplementary file 1 [file ijms-26-03480-s001.zip › ijms-3457485-supplementary.pdf]

Plasmon-Enhanced Visible and Near-Infrared Photodetector with Gold Nanorods Hybridized in a UCNPs/MoS_2 Device

Haitao Wei[a], Bowen Lv[a], Mengya Zhang[a], Xiangzhe Zhang[a], Xingheng Yan[b], Junhao Cai[a], Yaping Yang[b], Guomin Zhao[a], Tongcheng Yu[a*] and Kai Han[a*]

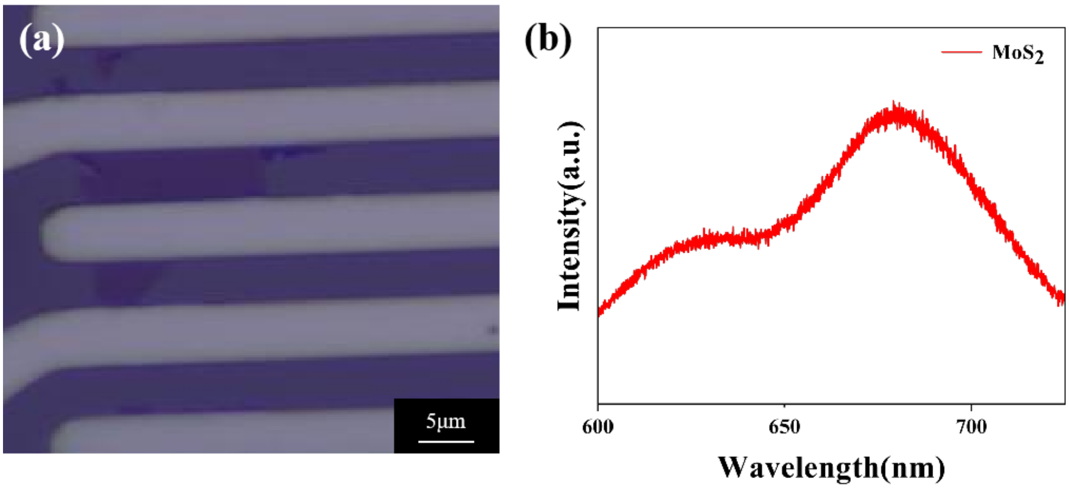

Fig.S1. (a) Photometric diagram of MoS_2. (b) PL spectrum of the MoS_2 flake on a SiO_2 substrate.

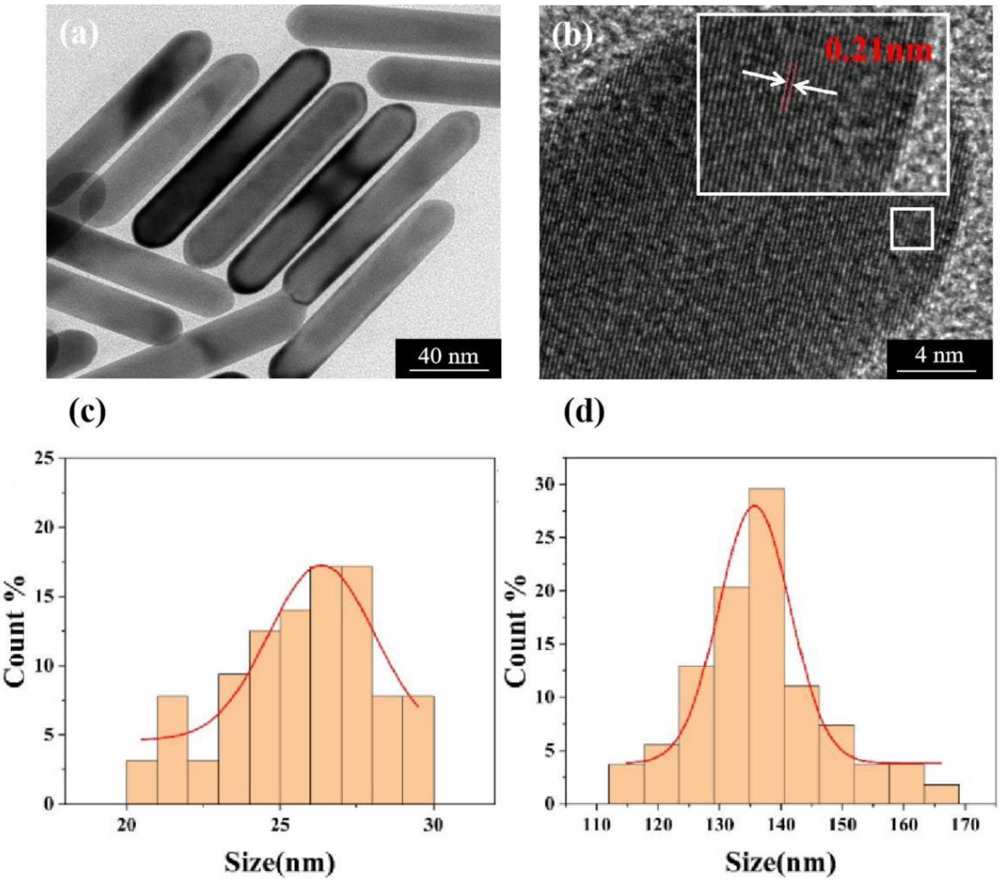

Fig.S2. (a) TEM image of Au NRs dispersed in water. (b) TEM image of Au NRs with a lattice spacing of 0.21 nm. (c) Size distribution of gold nanorods; the average particle size of gold nanorods is 25 nm. (d) The length distribution of Au NRs; the average length of the gold nanorods is 143 nm.

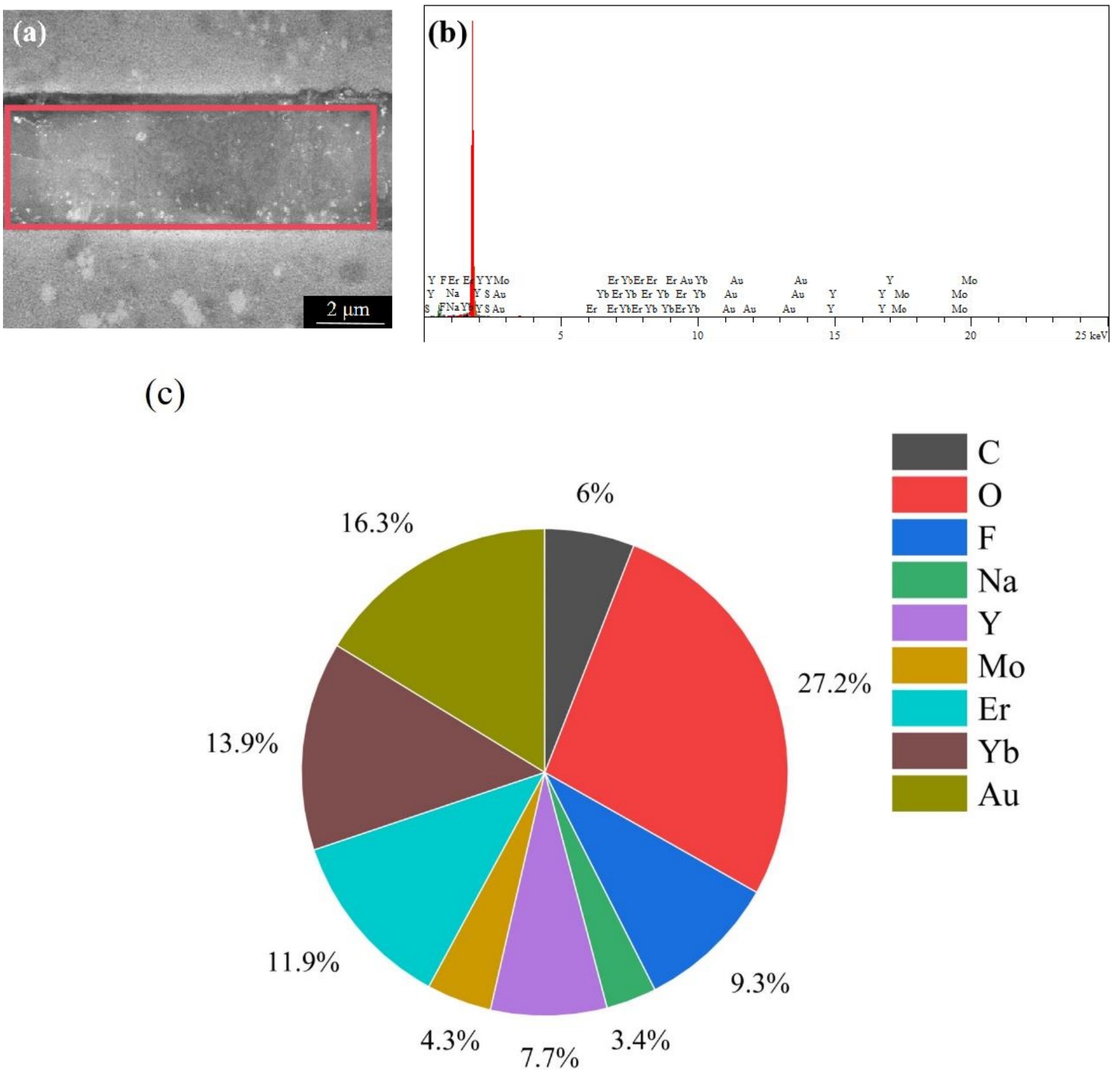

Fig.S3. (a) SEM image of the AuNRs/UCNPs/MoS_2 hybrid device. (b) EDS analysis diagram in the red area. (c) The content proportion of the elements in red area.

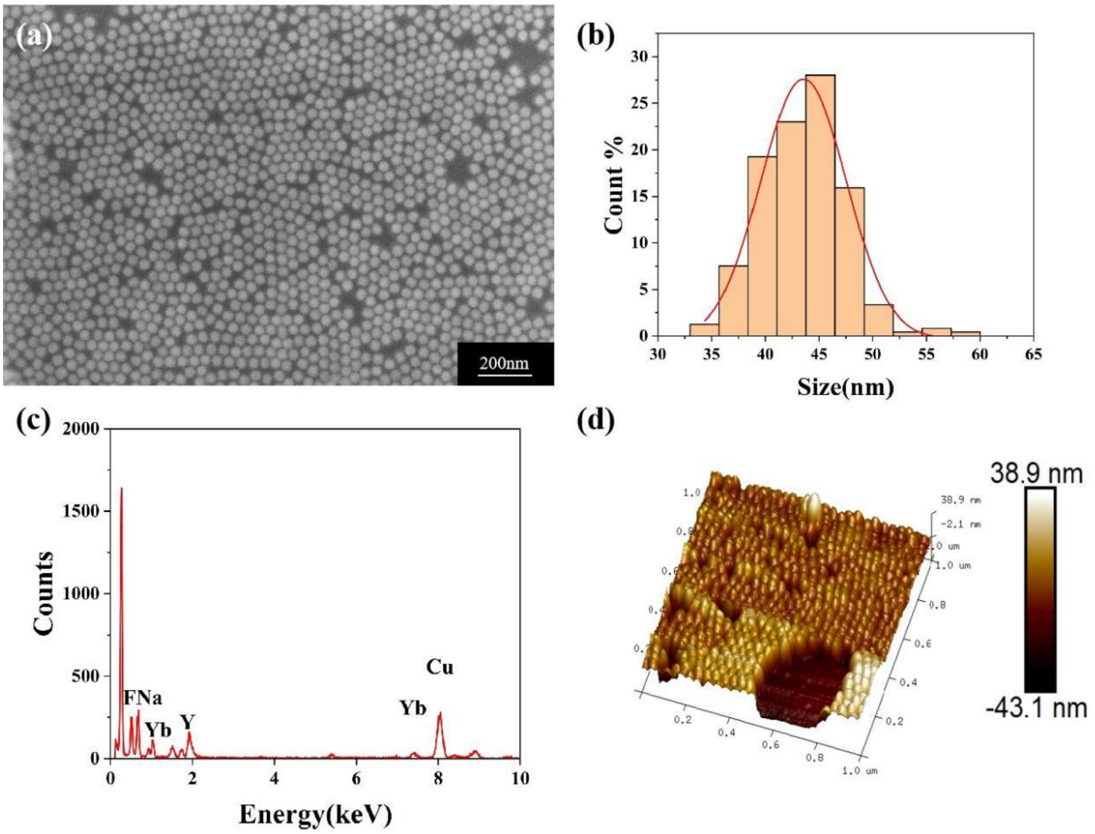

Fig.S4. (a) SEM image of UCNPs. (b) Particle size distribution diagram of UCNPs; the average diameter of a single particle is 43 nm. (c) EDX analysis diagram of UCNPs. (d) The thickness diagram of UCNPs was measured by AFM.

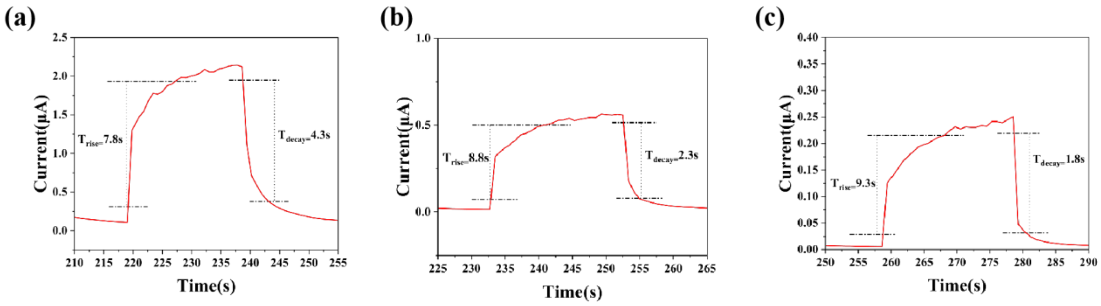

Fig.S5. (a) Time-resolved photocurrent rise and decay rate of the Au NRs/UCNPs/MoS_2 hybrid after 450 nm laser switching on and off under ambient conditions. (b) Time-resolved photocurrent rise and decay rate of the Au NRs/UCNPs/MoS_2 hybrid after 532 nm laser switching on and off under ambient conditions. (c) Time-resolved photocurrent rise and decay rate of the Au NRs/UCNPs/MoS_2 hybrid after 635 nm laser switching on and off under ambient conditions.

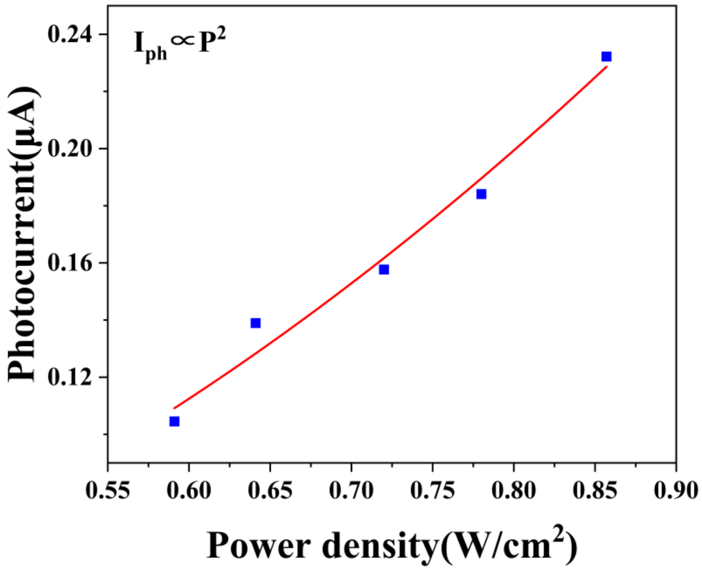

Fig.S6. Photocurrent of the Au NRs/UCNPs/MoS_2 hybrid device dependent on the intensity of illumination.

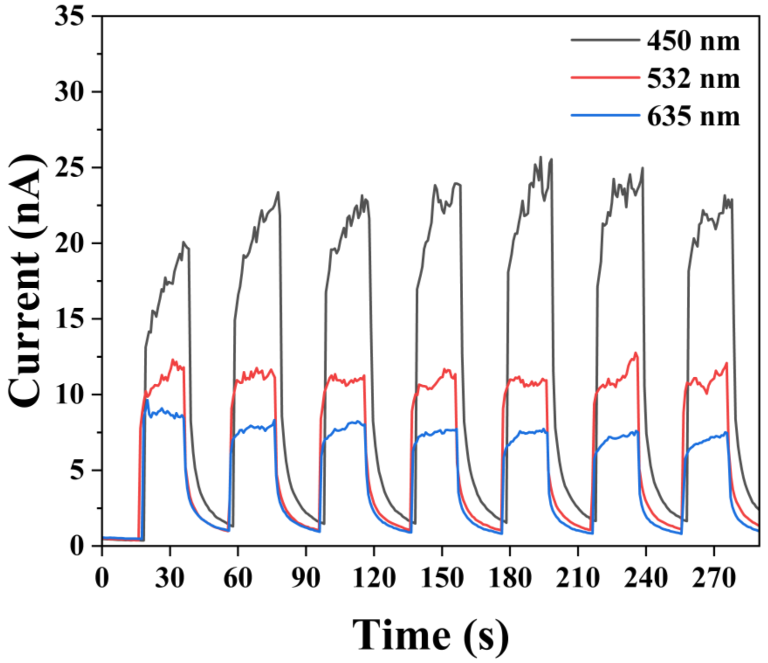

Fig.S7 Switching characteristic curves of the three devices under the same power density under 450, 532, 635 nm laser irradiation (P = 0.13W/cm^2).
